# Supplementary material for: Diet Pattern Analysis in Alzheimer’s Disease Implicates Gender Differences in Folate–B12–Homocysteine Axis on Cognitive Outcomes
Source: Nutrients. 2024 Mar 4;16(5):733. doi: 10.3390/nu16050733 (PMC10934747; doi:10.3390/nu16050733)
Supplement: Supplementary file 1 [file nutrients-16-00733-s001.zip › nutrients-2872773-supplementary.pdf]

**Supplementary File**

**Title:** Diet pattern analysis in Alzheimer disease implicates gender differences in folate-B12-homocysteine axis on cognitive outcomes

**Supplementary Table S1.** Blood profiles of the patients with Alzheimer disease

| | Total | | Male | | Female | | MW test |
|---|---|---|---|---|---|---|---|
| | Mean | SD | Mean | SD | Mean | SD | p-value |
| Homocysteine (μmol/L) | 12.31 | 4.74 | 13.72 | 5.21 | 11.34 | 4.14 | **<.001** |
| HS-CRP (mg/L) | 3.24 | 8.13 | 3.27 | 7.59 | 3.22 | 8.49 | 0.559 |
| Hemoglobin-A1C(%) | 6.12 | 0.90 | 6.12 | 0.88 | 6.13 | 0.92 | 0.845 |
| Fasting blood sugar(mg/dL) | 128.77 | 26.52 | 128.94 | 25.28 | 128.64 | 27.40 | 0.943 |
| Creatinine (mg/dL) | 1.01 | 0.82 | 1.12 | 0.55 | 0.94 | 0.96 | **<.001** |
| GOT (U/L) | 24.54 | 9.46 | 24.42 | 10.36 | 24.63 | 8.82 | 0.459 |
| GPT(U/L) | 21.23 | 12.39 | 22.70 | 13.32 | 20.22 | 11.62 | **0.044** |
| HDL-C(mg/dL) | 51.57 | 15.16 | 46.34 | 12.75 | 55.23 | 15.65 | **<.001** |
| VLDL-C(mg/dL) | 22.50 | 10.87 | 23.40 | 12.09 | 21.87 | 9.92 | 0.428 |
| LDL-C(mg/dL) | 107.12 | 34.55 | 103.27 | 30.80 | 109.81 | 36.76 | 0.085 |
| T-Cholesterol(mg/dL) | 182.17 | 39.09 | 173.37 | 33.55 | 188.35 | 41.50 | **<.001** |
| Triglyceride(mg/dL) | 117.20 | 72.83 | 119.40 | 67.66 | 115.67 | 76.32 | 0.541 |
| B12 (pg/mL) | 875.57 | 653.57 | 779.54 | 551.16 | 941.97 | 709.24 | **0.007** |
| Folate(pg/mL) | 13.70 | 8.90 | 11.87 | 8.09 | 14.96 | 9.22 | **<.001** |
| Albumin (g/dL) | 4.41 | 0.30 | 4.46 | 0.28 | 4.38 | 0.31 | **0.009** |
| Hemoglobin(g/dL) | 13.25 | 1.63 | 14.20 | 1.48 | 12.58 | 1.38 | **<.001** |

MW test: Mann-Whitney U test
SD: standard deviation; Comparisons between male and famale gender by Mann-Whitney U test

HS-CRP: high sensitive C reactive protein; HDL-C: high density lipoprotein C; VLDL-C: very low density lipoprotein C; LDL-C: Low density lipoprotein C; GOT: Glutamic Oxaloacetic Transaminase; GPT: Glutamic Pyruvic Transaminase

**Supplementary Table S2.** The regression analysis for MMSE score in patients with Alzheimer disease

|  | Coefficient | Std. error | t value | P value | 95% C.I. |  | VIF |
|---|---|---|---|---|---|---|---|
|  |  |  |  |  | Lower limit | Upper limit |  |
| Model for Male: adjusted R square=0.220; Durbin Watson's test statistic=1.643 |  |  |  |  |  |  |  |
| Constant | 14.583 | 6.339 | 2.301 | 0.023 |  |  |  |
| Age | -0.125 | 0.057 | -2.168 | 0.032* | -0.238 | -0.011 | 1.241 |
| Education | 0.297 | 0.12 | 2.477 | 0.014* | 0.06 | 0.533 | 1.234 |
| Body mass index | 0.355 | 0.133 | 2.672 | 0.008** | 0.093 | 0.617 | 1.061 |
| B12 | 0.003 | 0.001 | 3.233 | 0.001** | 0.001 | 0.004 | 1.006 |
| Coffee/ Tea | 0.596 | 0.223 | 2.666 | 0.008** | 0.155 | 1.036 | 1.069 |
| Model for Female: adjusted R square=0.224; Durbin Watson's test statistic=2.055 |  |  |  |  |  |  |  |
| Constant | 14.175 | 5.480 | 2.587 | .010 |  |  |  |
| Age | -.090 | .060 | -1.497 | .136 | -.208 | .028 | 1.205 |
| Education | .528 | .107 | 4.919 | <.001*** | .316 | .739 | 1.205 |
| Body mass index | .355 | .130 | 2.723 | .007** | .098 | .612 | 1.128 |
| Homocysteine | -.283 | .108 | -2.613 | .009** | -.496 | -.070 | 1.049 |
| Coffee/ Tea | .789 | .223 | 3.537 | <.001*** | .350 | 1.228 | 1.132 |

- *p<0.05, **p<0.01, ***P<0.001
- Dependent variables: mini-mental state examination (MMSE)
- Std. error: standard error
- C.I: confidence intervals
- VIF: variance inflation factor

**Figure Legend:**
**Supplementary Figure S1:**

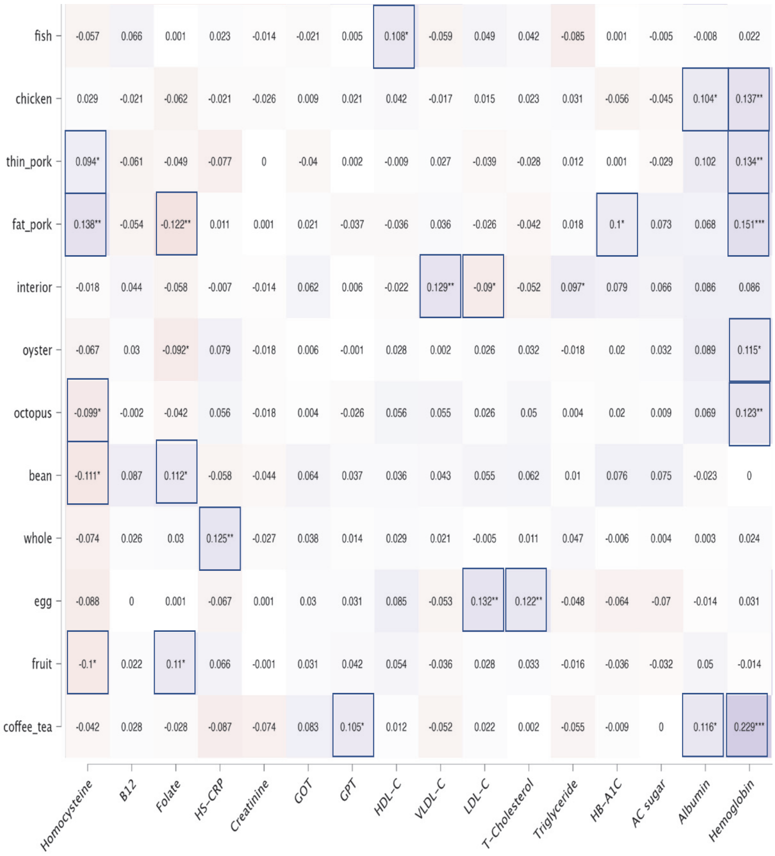


**Figure Legend:**
Correlation analysis heat map between blood profiles and diet pattern. Numbers indicate correlation coefficient. * p<0.05, **p<0.01, ***p<0.001

**Supplementary Figure S2:**

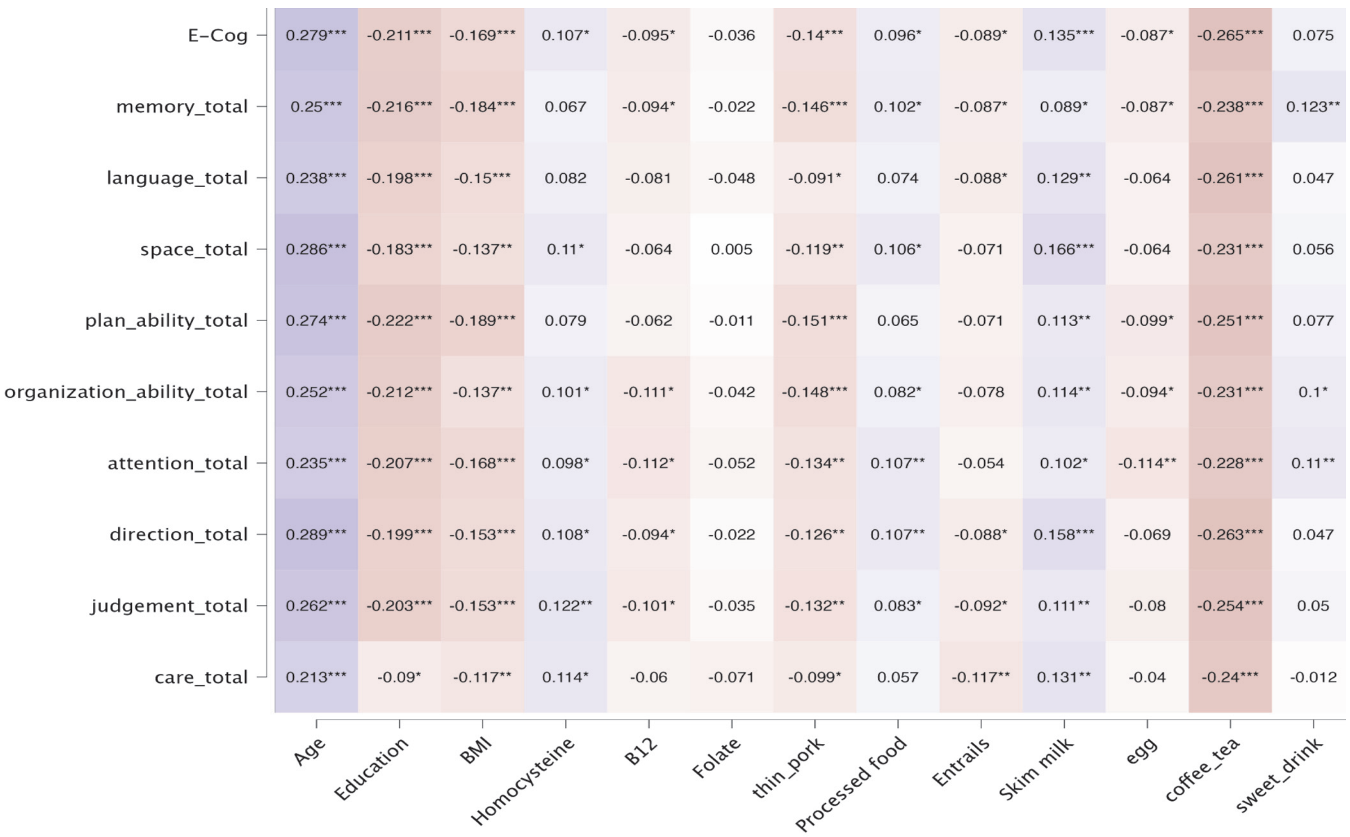


Figure Legend:
Correlation heat map between Y axis: Everyday cognition scale (and its subdomains) and X axis: demographic data, homocysteine-B12-folate axis, and diet pattern. The numbers indicated correlation coefficients. E-cog: Everyday cognition *p<0.05, **p<0.01, ***p<0.001

**Supplementary Figure S3:**

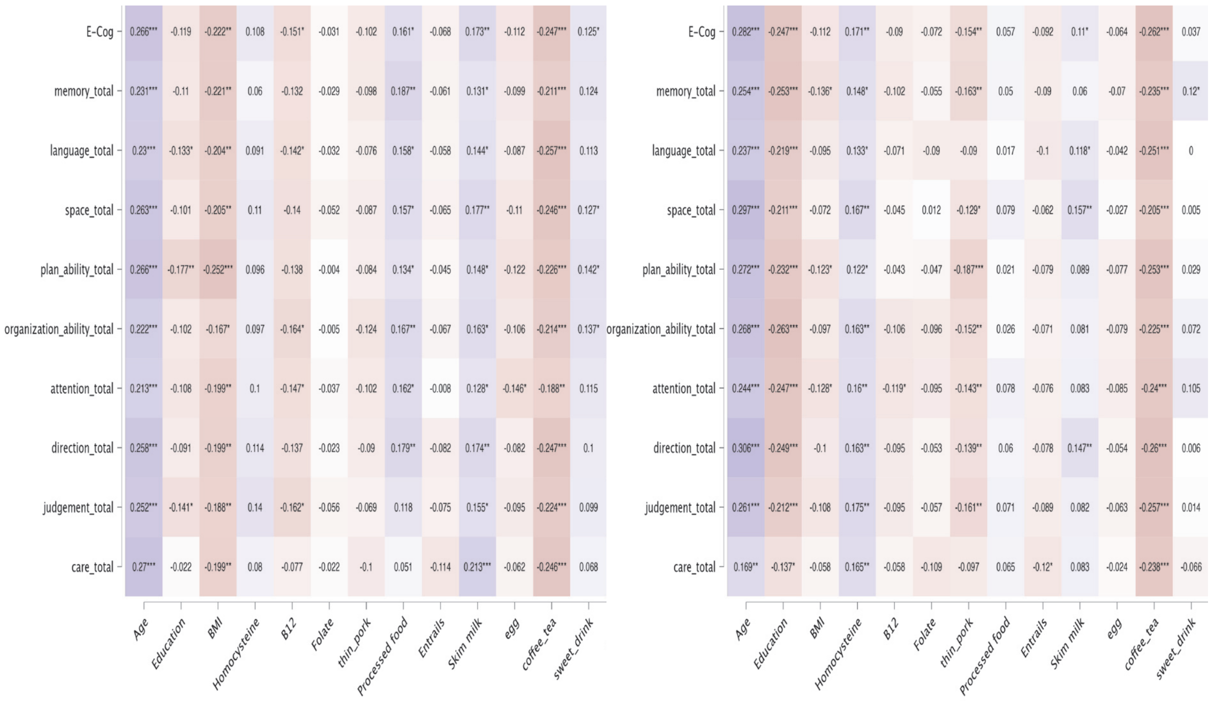

Male patient                                  Female patient

Figure Legend: Gender effect of homocysteine and B12 on Every-day Cognition (E-Cog). Correlation heat map between Y axis: Everyday cognition scale (and its subdomains) and X axis: demographic data, homocysteine-B12-folate axis, and diet pattern. The numbers indicated correlation coefficients.
*: p-value < 0.05; **: p-value < 0.01; ***: p-value < 0.001.
